# Use of receptor and nonreceptor tyrosine kinase inhibitors and Parkinson disease risk

**DOI:** 10.1016/j.neurot.2026.e01040

**Published:** 2026-08-27

**Authors:** Giovanni R. Malaty, Jordan A. Killion, Sai Anmisha Doddamreddy, Osvaldo J. Laurido-Soto, Brad A. Racette

**Affiliations:** a Department of Neurology, Barrow Neurological Institute, St. Joseph's Hospital and Medical Center, Phoenix, AZ, USA; b Department of Neurology, Washington University School of Medicine in St. Louis, St. Louis, Missouri, USA; c Center for Advancing Health Services, Policy & Economics Research, Washington University, St. Louis, Missouri, USA; d School of Public Health, University of the Witwatersrand, Johannesburg, South Africa

**Keywords:** Nonreceptor tyrosine kinase inhibitors, Parkinson disease, Pharmacoepidemiology, Receptor tyrosine kinase inhibitors

## Abstract

There are currently no disease-modifying therapies for Parkinson disease (PD), although various targets for disease modification have been explored. Expression and binding of receptor tyrosine kinases (rTKs) and nonreceptor tyrosine kinases (nrTKs) have been implicated in PD pathogenesis, and their inhibition is a promisingneuroprotective strategy. This population-based, case-control study of US Medicare beneficiaries included 207,532 incident PD patients and 975,177 comparable, population-based control subjects from 2016 to 2018. This study investigated associations between Medicare Part D prescription fills prior to PD diagnosis for 7 rTK inhibitors (erlotinib, sorafenib, pazopanib, imatinib, sunitinib, nintedanib, and dasatinib) and 4 nrTK inhibitors (nilotinib, ibrutinib, ruxolitinib, and tofacitinib) and PD risk. Logistic regression estimated relative risks (RR) and 95% confidence intervals (CIs) adjusted for age, sex, race, smoking, and healthcare utilization. All medications were inversely associated with incident PD. The rTK inhibitors erlotinib (RR = 0.53, 95% CI: 0.38–0.74), sorafenib (RR = 0.43, 95% CI: 0.25–0.74), imatinib (RR = 0.60, 95% CI: 0.47–0.75), pazopanib (RR = 0.58, 95% CI: 0.39–0.86), nintedanib (RR = 0.66, 95% CI: 0.50–0.88), sunitinib (RR = 0.60, 95% CI: 0.39–0.93), and dasatinib (RR = 0.53, 95% CI: 0.35–0.82) had the strongest inverse associations. Among nrTK inhibitors, ibrutinib (RR = 0.62, 95% CI: 0.51–0.75) had a marked inverse association. Given the chemotherapeutic mechanisms of these medications, and the known cancer–PD inverse relationship, we performed a sensitivity analysis adjusting for relevant cancer subtypes, yielding a similar inverse association. Overall, the use of rTK and nrTK inhibitors was associated with a lower risk of developing PD, although there may be differential impact of this medication class depending on specific inhibition pathways.

## Introduction

The pathophysiologic hallmark of Parkinson disease (PD) is progressive reduction in dopamine-producing neurons in the substantia nigra and the nigrostriatal pathway. Although the misfolding and pathologic aggregation of alpha-synuclein protein is thought to represent the primary process driving cell death and neurodegeneration in PD, other co-occurring pathogenic processes likely lead to neuronal degeneration, including neuronal mitochondrial dysfunction, defects in proteolysis, abnormal iron metabolism, oxidative stress, and neuroinflammation [[1], [2], [3], [4], [5], [6], [7], [8], [9], [10]]. Pharmacologic management in PD has historically centered around improving motor symptoms via dopamine replacement and various adjunctive medications. At present, no disease-modifying treatments exist for the management of PD, and clinical trials of medications targeting disease progression have been mostly unsuccessful [[11], [12], [13], [14], [15], [16], [17], [18], [19], [20], [21], [22], [23], [24]].

The two subtypes of protein kinases, receptor tyrosine kinases (rTKs) and nonreceptor tyrosine kinases (nrTKs), are potential therapeutic targets in PD given their wide distribution throughout the central nervous system (CNS; including the basal ganglia), extensively documented involvement in the CNS signaling pathways, and potential role in neurodegeneration [25,26]. Typically, the upregulation of several rTKs located in the CNS is associated with neuroprotective, neurotrophic, and neuron-sparing effects. However, several of these same kinases have also been contradictorily associated with CNS neurotoxic effects, depending on their levels of expression and the context of ongoing disease states, including PD [[27], [28], [29], [30], [31]]. Overexpression of nrTKs almost uniformly results in pathologic neuroinflammation and neurodegeneration in PD models, whereas their inhibition confers neuroprotection [[32], [33], [34]].

Several studies aiming to repurpose rTK and nrTK inhibitors for the treatment of PD have been conducted, with mostly mixed results. Most notably, nilotinib, an oral inhibitor of the nrTK cellular Abelson murine leukemia viral oncogene homolog 1 (cAbl), approved for the treatment of chronic myeloid leukemia, was tested in multiple single-center and multicenter randomized control trials for tolerability and safety, and efficacy in patients with PD [18,35]. Although nilotinib demonstrates good tolerability and safety at several different doses, there is minimal evidence of disease modification. A study of nilotinib in patients with dementia with Lewy bodies (DLB), however, has demonstrated favorable safety, biomarker, and efficacy outcomes [36,37]. More recent results regarding similar medications are mixed. The nrTK (cAbl/SRC) inhibitor Bosutinib, for example, shows promise in DLB and Alzheimer's disease (AD), while the potent cAbl inhibitor, Vodobatinib failed to demonstrate target engagement or disease modification in either PD or DLB [[38], [39], [40], [41]].

Nevertheless, the broader category of protein kinase inhibitors remains promising because unique pharmacologic profiles may demonstrate differential neuroprotective effects in PD. To explore this relationship, we sought to test the association between the use of 11 different rTK and nrTK inhibitors (Table 1 and Table 2) and PD risk using population-based US Medicare data.

**Table 1:** Specific mechanisms of inhibition of selected rTK and nrTK inhibitors.

Inhibitor	Mechanism(s) of inhibition	
	rTK	nrTK
rTK inhibitors		
Erlotinib	EGFR	–
Dasatinib	PDGF, Eph	ABL, TEC, SRC, BTK
Sorafenib	VEGF, PDGF	–
Pazopanib	FGFR, VEGF, PDGF	–
Imatinib	PDGF	ABL
Sunitinib	VEGF, PDGF	–
Nintedanib	FGFR, VEGF, PDGF	–
nrTK inhibitors		
Nilotinib	PDGF	ABL
Ibrutinib	–	BTK
Ruxolitinib	–	JAK
Tofacitinib	–	JAK

Abbreviations: ABL, Ableson murine leukemia; BTK, Bruton's tyrosine kinase; EGFR, epidermal growth factor receptor; Eph, Ephrin receptor-binding domain; FGFR, fibroblast growth factor receptor; JAK, Janus kinase; nrTK, nonreceptor tyrosine kinase; PDGF, platelet-derived growth factor; rTK, receptor tyrosine kinase; SRC, proto-oncogene tyrosine-protein kinase Src; TEC, Tec protein tyrosine kinase; VEGF, vascular endothelial growth factor.

**Table 2:** Labeled Indications of selected rTK and nrTK inhibitors.

Medication	Indication
**rTK inhibitors**	
Erlotinib	NSCLC, Pancreatic cancer
Sorafenib	HCC, RCC, differentiated thyroid carcinoma
Pazopanib	RCC, STS
Imatinib	CML, BCR/ABL-positive, ALL-Ph+, GIST, MDS/MPD with PDGFR rearrangement, ASM, HES, CEL, DFSP
Sunitinib	GIS, RCC, pNET
Nintedanib	IPF, Chronic fibrosing ILD, Systemic sclerosis-associated ILD
Dasatinib	CML, ALL
**nrTK inhibitors**	
Nilotinib	CML, SLL, CLL
Ibrutinib	CLL, SLL, Waldenström's macroglobulinemia, cGVHD
Ruxolitinib	Primary myelofibrosis, Post-PV myelofibrosis, Post-essential thrombocythemia myelofibrosis, PV, aGVHD/cGVHD
Tofacitinib	RA, PsA, AS, UC, JIA

NSCLC, non-small cell lung cancer; HCC, hepatocellular carcinoma; RCC, renal cell carcinoma; STS, soft tissue sarcoma; CML, Chronic myeloid leukemia; BCR/ABL, Breakpoint Cluster Region-Abelson; -positive; ALL, acute lymphoblastic leukemia; Ph+, Philadelphia chromosome positive; GIST, gastrointestinal stromal tumor; MDS/MPD, myelodysplastic syndrome/myelodysplasia; PDGFR, platelet-derived growth factor receptor; ASM, aggressive systemic mastocytosis; HES, Hypereosinophilic syndrome; CEL, Chronic eosinophilic leukemia; DFSP, Dermatofibrosarcoma protuberans; pNET, pancreatic neuroendocrine tumor; IPF, idiopathic pulmonary fibrosis; ILD, interstitial Lung disease; SLL, small lymphocytic lymphoma; CLL, Chronic lymphocytic leukemia; cGVHD; Chronic graft-versus-host disease; aGVHD; acute graft-versus-host disease; PV, Polycythemia vera; RA, Rheumatoid arthritis; PsA Psoriatic arthritis; AS, Ankylosing spondylitis; UC, ulcerative colitis; JIA, juvenile idiopathic arthritis.

## Materials and methods

### Human subjects

This study was determined to be exempt by the Human Research Protection Office at Washington University School of Medicine in St. Louis, Barrow Neurological Institute, and the Centers for Medicare & Medicaid Services.

### Study design

We performed a population-based case-control study of incident PD cases and controls from 2016 to 2018 that used Medicare beneficiary data from the period 2014–2018. To ascertain case and control status and to obtain clinical and demographic information, we used Medicare carrier (physician or supplier Part B), outpatient, inpatient, skilled nursing facility, durable medical equipment, home health care claims, beneficiary annual summary file, and Medicare Part D claims data. Inclusion in the study for both cases and controls required beneficiaries to be Medicare age-eligible for 2 or more years prior to their diagnosis or control reference date (age ≥67 years); be less than 111 years old; have coverage under Medicare Parts A, B, and D at the time of case diagnosis or control selection; have US residence; and lack coverage by Medicare Advantage plan (Part C).

### Ascertainment of cases and controls

We used comprehensive Part A and B claims data from 2014 to 2016 to determine incident PD status. Beneficiaries with at least 1 diagnosis code of PD (International Classification of Diseases, Tenth Revision, Clinical Modification [ICD-10-CM]: G20) during 2016–2018 and no PD diagnosis before 2016 (ICD-10-CM: G20 or International Classification of Diseases, Ninth Revision, Clinical Modification [ICD-9-CM]: 332.0) were identified as incident PD cases. Those with a history of DLB (ICD-9-CM: 331.82 or ICD-10-CM: G31.83) or atypical parkinsonism (ICD-9-CM: 333.0 or ICD-10-CM: G23.1, G23.2, G23.8, G23.9, or G90.3) were excluded from the primary analysis.

Medicare beneficiaries meeting inclusion criteria without a documented history of PD, DLB, or atypical parkinsonism were selected at random with a 5:1 ratio of controls to cases based on the month and year of PD diagnosis. Although controls were matched for a larger study before implementation of the Part D coverage requirement, we maintained a match ratio of greater than 4:1 controls to cases after requiring Part D coverage at the time of PD diagnosis or control selection date. Controls were not matched to cases on the basis of race, sex, age, or other covariates; however, we adjusted for these variables in analysis.

As an exploratory analysis, we identified beneficiaries with incident DLB cases during 2016–2018 and with no DLB diagnosis before 2016 using the same methodology described for PD cases and excluding any beneficiary with a history of PD or atypical parkinsonism. Controls for the DLB cases were selected using a 5:1 ratio based on the month and year of diagnosis using the same exclusion and enrollment criteria described above.

### Ascertainment of exposure status

Medicare Part D data from 2014 through 2018 were used to identify rTK and nrTK inhibitor prescriptions as binary variables (yes/no) indicating whether the beneficiary filled those medications before the date of diagnosis or control selection. We categorized erlotinib, sorafenib, pazopanib, imatinib, sunitinib, nintedanib, and dasatinib as rTK inhibitors. We categorized nilotinib, ibrutinib, ruxolitinib, and tofacitinib as nrTK inhibitors. Over the counter medications and nonsystemic formulations (e.g., topical and ophthalmologic, as in the case of Janus kinase (JAK) inhibitors) were excluded from analysis.

### Covariates

We adjusted *a priori* for Medicare data-derived variables including age (continuous), sex (male or female), and race or ethnicity (non-Hispanic White, Black, Hispanic, Asian/Pacific Islander, Native American, and other), smoking status (continuous variable of the estimated probability of having ever smoked) [42], and a measure of healthcare utilization (continuous variable of the number of unique diagnosis codes) [43]. Age, sex, and race data were obtained from the Medicare beneficiary summary file [42]. Smoking status was predicted using a validated algorithm utilizing Medicare claims data. Healthcare utilization [43] was captured from Medicare claims data and taken up to the PD diagnosis or control reference date for the unlagged models; for the lagged model, this variable was captured up to the lagged exposure date.

Furthermore, we identified cancer ICD-10 codes and other diagnoses of interest prior to the PD or DLB diagnosis or control reference date back to 2014. Cancer and other diagnoses were classified into four clinically meaningful categories: (1) solid tumors and solid organ malignancies, (2) hematologic malignancies and myeloproliferative disorders, (3) immune-mediated, autoimmune, and inflammatory disorders, and (4) fibrotic interstitial lung diseases. We created a binary cancer variable for these cancer diagnoses of interest prior to PD diagnosis or control reference date which we included as a covariate in a sensitivity model. Age at first cancer diagnosis was calculated as the difference between the date of earliest diagnosis date for a cancer of interest and the beneficiary's date of birth, expressed in years.

### Statistical analysis

Using Stata version 17.0 (StataCorp/MP, College State, TX), we performed logistic regressions to calculate the odds ratio as an estimate of the relative risk (RR) and 95% confidence intervals (CIs) for each medication as the independent variable and PD as the outcome, adjusting *a priori* for age (continuous), sex (male or female), race or ethnicity (6 categories), smoking status (continuous), and healthcare utilization (continuous). As a sensitivity analysis, we ran all models with a 2-year exposure lag. The 2-year lagged models included only beneficiaries who filled a prescription using Medicare Part D within the 2 years before their PD diagnosis or control reference date. We repeated the same analyses for DLB cases and controls. All models were restricted to those with 11 or more cases and 11 or more controls as per Centers for Medicare and Medicaid Services reporting requirements.

We then conducted a sensitivity analysis examining the proportion of days covered (PDC) across a two-year timespan for each of the 11 TKIs. We first restricted the sample to beneficiaries with continuous Part D prescription drug coverage for a full two years prior to diagnosis to ensure a fixed 730-day observation window. The PDC was calculated for each drug as the proportion of days that the medication was filled over the two-year window prior to diagnosis or control reference date, with PDC values ranging from 0 (no fills) to 1 (fully covered). Logistic regression models were run for each drug using PDC as a continuous exposure variable, adjusted for age, sex, race/ethnicity, smoking probability, and healthcare utilization. The models were also re-run including an additional adjustment variable for if the beneficiary had a cancer diagnosis of interest prior to diagnosis or control reference date (yes/no). PDC analyses were conducted for both outcomes of PD and DLB, however the PDC results for DLB cases are not able to reported given the CMS guidelines for small sample sizes.

## Results

We included 207,532 incident PD cases and 975,177 population-based controls in this case-control study (Table 3). PD cases were slightly older, were more likely to be male, were less likely to have ever smoked, and had greater healthcare utilization than controls. For our exploratory analysis, we included 32,853 incident DLB cases and 164,255 controls (Table 2). DLB cases were slightly older, were more likely to be female, had a higher smoking index, and had greater healthcare utilization than controls.

**Table 3:** Characteristics of incident Parkinson disease (PD) and dementia with Lewy bodies (DLB) cases and controls using 2016–2018 U.S. Medicare data.

	PD analysis		DLB analysis	
	PD Cases (n = 207,532) %	Controls (n = 975,177) %	DLB Cases (n = 32,853) %	Controls (n = 164,255) %
**Sex**				
Male	50.7	39.9	42.7	39.9
Female	49.3	60.1	57.3	60.1
**Age**				
Mean (SD)	78.8 (7.3)	75.8 (7.4)	81.3 (7.6)	75.9 (7.7)
Median (Range)	78 (67–109)	74 (67–110)	81 (67–109)	74 (66–110)
**Race/Ethnicity**				
Non-Hispanic White	83.9	83.3	83.4	82.0
Black	5.9	7.1	8.2	7.8
Hispanic	6.0	5.3	5.4	5.8
Asian/Pacific Islander	3.2	3.2	2.1	3.3
Native American	0.3	0.4	0.3	0.4
Other	0.7	0.7	0.6	0.8
**Smoking Index**^a^	34.0	48.8	53.6	49.3
**Healthcare Utilization**^b^				
Mean (SD)	108.4 (67.7)	73.1 (52.6)	112.4 (64.7)	72.9 (53.5)
Median (Range)	97.0 (0–650)	61.0 (0–630)	101.0 (0–603)	61.0 (0–528)
**Cancer Prior to PD diagnosis**				
**Any cancer of interest**				
Yes	9.3	7.0	8.6	7.3
No	90.7	93.0	91.4	92.7
Age at cancer diagnosis^c^				
Mean (SD)	77.5 (7.1)	74.9 (7.2)	79.9 (7.4)	75.3 (7.6)
Median (Range)	77 (63–104)	74 (63–107)	80 (64–103)	74 (63–106)
**Solid Tumors**^d^				
Yes	2.6	2.2	2.2	2.4
No	97.4	97.8	97.8	97.6
Age at cancer diagnosis^c^				
Mean (SD)	76.8 (6.8)	74.6 (6.8)	79.1 (7.1)	75.1 (7.2)
Median (Range)	76 (64–103)	73 (63–104)	78 (64–100)	74 (63–101)
**Hematologic Malignancies**^e^				
Yes	1.6	1.2	1.6	1.2
No	98.4	98.8	98.4	98.8
Age at cancer diagnosis^c^				
Mean (SD)	78.8 (7.2)	76.4 (7.6)	81.3 (7.2)	76.9 (7.9)
Median (Range)	78 (64–104)	75 (63–105)	82 (65–100)	76 (63–106)
**Immune-Mediated/Autoimmune**^f^				
Yes	5.2	3.7	4.9	3.8
No	94.8	96.3	95.1	96.2
Age at cancer diagnosis^c^				
Mean (SD)	77.3 (7.1)	74.6 (7.2)	79.6 (7.5)	74.7 (7.5)
Median (Range)	77 (63–101)	73 (63–107)	80 (64–103)	73 (63–105)
**Fibrotic Malignancies**^g^				
Yes	0.3	0.3	0.3	0.3
No	99.7	99.7	99.7	99.7
Age at cancer diagnosis^c^				
Mean (SD)	78.9 (7.2)	76.8 (7.3)	81.1 (7.3)	78.0 (7.6)
Median (Range)	79 (64–98)	76.0 (64–100)	81 (66–96)	78 (63–98)

Abbreviations: PD, Parkinson disease; U.S., United States; SD, standard deviation.

a Percentage less than the median of the estimated probability of ever smoking divided by the number of unique diagnosis codes (or by 1 if no codes).

b Number of unique diagnosis codes prior to PD diagnosis or control reference date.

c Age at cancer diagnosis for selected cancers only among those with a cancer diagnosis.

d Diagnoses include non-small cell lung cancer (NSCLC), pancreatic adenocarcinoma, hepatocellular carcinoma (HCC), renal cell carcinoma (RCC), differentiated thyroid carcinoma (DTC), gastrointestinal stromal tumors (GIST), soft tissue sarcomas (STS), dermatofibrosarcoma protuberans (DFSP), pancreatic neuroendocrine tumor (pNET).

e Diagnoses include chronic myeloid leukemia (CML), Philadelphia chromosome-positive acute lymphoblastic leukemia (Ph + ALL), chronic lymphocytic leukemia (CLL), small lymphocytic lymphoma (SLL), Waldenström macroglobulinemia, Myelodysplastic/myeloproliferative neoplasms (MDS/MPD), aggressive systemic mastocytosis, chronic eosinophilic leukemia, hypereosinophilic syndrome, primary myelofibrosis, post-polycythemia vera myelofibrosis, post-essential thrombocythemia myelofibrosis, Polycythemia vera.

f Diagnoses include rheumatoid arthritis, psoriatic arthritis, ankylosing spondylitis, ulcerative colitis, juvenile idiopathic arthritis, acute graft-versus-host disease, chronic graft-versus-host disease.

g Diagnoses include idiopathic pulmonary fibrosis (IPF), progressive fibrosing interstitial lung disease (PF-ILD), systemic sclerosis-associated ILD.

All rTK inhibitors assessed had an inverse association with PD risk (Table 4). Sorafenib was associated with the lowest PD risk (RR = 0.43, 95% CI: 0.25–0.74), followed by erlotinib (RR = 0.51, 95% CI: 0.38–0.74) and dasatinib (RR = 0.53, 95% CI: 0.35–0.82). With a 2-year lag, beneficiaries receiving erlotinib (RR = 0.54, 95% CI: 0.34–0.85) and dasatinib (RR = 0.48, 95% CI: 0.27–0.84) still had substantially lower risk of developing PD (Table 4). The RR associated with sorafenib with a 2-year lag could not be calculated due to the small sample size. Each nrTK inhibitor examined also had an inverse association with PD, with ibrutinib associated with the lowest RR at 0.62 (95% CI: 0.51–0.75), followed by nilotinib (RR = 0.68, 95% CI: 0.45–1.02). With a 2-year lag, we still observed an inverse relation between ibrutinib (RR = 0.49, 95% CI: 0.34–0.72) and nilotinib (RR = 0.39, 95% CI 0.21–0.74) and PD risk (Table 5).

**Table 4:** Receptor and nonreceptor tyrosine kinase inhibitors and Parkinson disease (PD) and dementia with Lewy bodies (DLB) risk among US Medicare beneficiaries, 2016–2018.

Medication	PD cases (n = 207,532)	Controls (n = 975,177)	RR (95% CI)^a^	DLB cases (n = 32,853)	Controls (n = 164,255)	RR (95% CI)^a^
rTK inhibitors						
Erlotinib	41	279	**0.53 (0.38–0.74)**	–	–	–
Sorafenib	17	88	**0.43 (0.25–0.74)**	–	–	–
Pazopanib	32	157	**0.58 (0.39–0.86)**	–	–	–
Imatinib	95	490	**0.60 (0.47–0.75)**	12	74	0.57 (0.30–1.08)
Sunitinib	28	123	**0.60 (0.39–0.93)**	–	–	–
Nintedanib	61	287	**0.66 (0.50–0.88)**	–	–	–
Dasatinib	27	147	**0.53 (0.35–0.82)**	–	–	–
nrTK inhibitors						
Nilotinib	31	130	0.68 (0.45–1.02)	–	–	–
Ibrutinib	131	550	**0.62 (0.51–0.75)**	12	104	**0.36 (0.20–0.67)**
Ruxolitinib	63	239	0.79 (0.59–1.06)	–	–	–
Tofacitinib	83	320	0.89 (0.69–1.16)	–	–	–

Boldface type indicates statistical significance (p < 0.05). Abbreviations: CI, confidence interval; DLB, dementia with Lewy bodies; nrTK, nonreceptor tyrosine kinase; PD, Parkinson disease; RR, relative risk; rTK, receptor tyrosine kinase.

a RR adjusted for age (continuous), sex, race or ethnicity, smoking, and healthcare utilization.

**Table 5:** Receptor and nonreceptor tyrosine kinase inhibitors and Parkinson disease (PD) risk among US Medicare beneficiaries, 2016–2018, with a 2-year lag.^a^

Medication	PD cases (n = 184,164)	Controls (n = 816,842)	RR (95% CI)^b^
**rTK inhibitors**			
Erlotinib	22	147	**0.54 (0.34–0.85)**
Sorafenib^c^	–	–	**-**
Pazopanib	15	55	0.72 (0.40–1.29)
Imatinib	70	354	**0.61 (0.47–0.80)**
Sunitinib	12	49	**0.51 (0.27–1.00)**
Nintedanib^c^	–	–	**-**
Dasatinib	15	93	**0.48 (0.27–0.84)**
**nrTK inhibitors**			
Nilotinib	12	85	**0.39 (0.21–0.74)**
Ibrutinib	35	175	**0.49 (0.34–0.72)**
Ruxolitinib	26	104	0.72 (0.45–1.13)
Tofacitinib	45	138	1.04 (0.73–1.50)

Boldface type indicates statistical significance (p < 0.05).

**Abbreviations:** CI, confidence interval; nrTK, nonreceptor tyrosine kinase; PD, Parkinson disease; RR, relative risk; rTK, receptor tyrosine kinase.

a Restricted to cases and controls who filled a prescription using Medicare Part D within the 2 years prior to PD diagnosis or control reference date.

b RR adjusted for age (continuous), sex, race or ethnicity, smoking, and healthcare utilization.

c Suppressed due to small numbers (<11 cases or controls).

In our exploratory analysis, we evaluated the association between these medications and incident DLB. Due to small sample sizes, we could estimate the risk of DLB only in relation to ibrutinib and imatinib. Ibrutinib (RR = 0.36, 95% CI: 0.20–0.67) and imatinib (RR = 0.57, 95% CI: 0.30–1.08) were both inversely associated with DLB risk. With a 2-year lag, small counts precluded the reporting of any associations.

In the PDC analyses, all rTK inhibitors still had point estimates that were inversely associated with PD, but sorafenib, pazopanib, and sunitinib now cross the null (Table 6). Adjusting for a cancer of interest prior to PD diagnosis attenuated the results slightly toward the null but did not change the findings. All four nrTK inhibitors still had point estimates that indicated an inverse association with PD in the PDC analysis, but ibrutinib was still the only one that did not cross the null, and this remained after adjusting for a prior cancer of interest. We were unable to show results for the PDC calculations for DLB cases due to small sample sizes.

**Table 6:** Proportion of days covered (PDC) for receptor and non-receptor tyrosine kinase inhibitors and Parkinson disease (PD) risk among U.S. Medicare beneficiaries 2016–2018.

Medication	PD cases (n = 188,513)	Controls (n = 879,753)	Median PDC (IQR)	RR (95% CI)^a^	RR (95% CI)^b^
***rTK inhibitors***					
Erlotinib	35	225	0.41 (0.16–0.77)	**0.41 (0.21–0.83)**	**0.48 (0.24–0.96)**
Sorafenib	14	70	0.12 (0.04–0.28)	0.31 (0.04–2.21)	0.40 (0.06–2.74)
Pazopanib	28	124	0.21 (0.08–0.41)	0.40 (0.12–1.31)	0.50 (0.15–1.59)
Imatinib	82	409	0.74 (0.39–0.94)	**0.59 (0.42–0.85)**	**0.66 (0.46–0.95)**
Sunitinib	22	87	0.15 (0.08–0.35)	0.40 (0.08–2.03)	0.50 (0.10–2.51)
Nintedanib	53	251	0.25 (0.11–0.57)	**0.27 (0.12–0.60)**	**0.32 (0.15–0.71)**
Dasatinib	21	120	0.45 (0.16–0.82)	**0.32 (0.13–0.81)**	**0.38 (0.15–0.94)**
***nrTK inhibitors***					
Nilotinib	29	106	0.73 (0.31–0.93)	0.59 (0.31–1.12)	0.67 (0.35–1.27)
Ibrutinib	115	490	0.37 (0.12–0.71)	**0.47 (0.31–0.72)**	**0.54 (0.36–0.82)**
Ruxolitinib	63	217	0.54 (0.16–0.91)	0.81 (0.50–1.30)	0.92 (0.57–1.48)
Tofacitinib	61	235	0.25 (0.12–0.60)	0.73 (0.37–1.41)	0.85 (0.44–1.65)

Bold indicates statistical significance (p < 0.05).

**Abbreviations:** PD, Parkinson disease; U.S., United States; CI, confidence interval; rTK, receptor tyrosine kinase; nrTK, non-receptor tyrosine kinase; PDC, proportion of days covered; IQR, interquartile range.

a Odds ratio adjusted for age (continuous), sex, race/ethnicity, smoking, and healthcare utilization, restricted to beneficiaries with continuous Part D coverage for 2 years prior to diagnosis.

b Odds ratio adjusted for age (continuous), sex, race/ethnicity, smoking, healthcare utilization and cancer restricted to beneficiaries with continuous Part D coverage for 2 years prior to diagnosis.

## Discussion

In this administrative data study of the use of rTK and nrTK inhibitors, we found an inverse association between the use of all 11 rTK and nrTK inhibitors and incident PD, and this inverse relationship was still seen for most medications with a 2-year exposure lag, as well as when adjusting for specific relevant cancer and other diagnoses. Additionally, we saw an inverse relationship with the two medications (ibrutinib and imatinib) for which we had sufficient sample sizes to examine possible associations with DLB risk. Our study is consistent with literature that suggests the potentially deleterious effects of pathologically upregulated and overexpressed rTKs on the brain, including epidermal growth factor receptor (EGFR), vascular endothelial growth factor (VEGF), and proto-oncogene B-Raf, because their inhibition appeared to lower the risk of developing PD. Similarly, the increased expression of the nrTKs, including Bruton's tyrosine kinase (BTK), cellular Abelson murine leukemia viral oncogene homolog 1 (cAbl), proto-oncogene tyrosine-protein kinase Src (SRC), and JAK, in the CNS has routinely been associated with inflammation and neurodegeneration. These proteins have been the targets of preclinical studies and clinical trials as potential disease-modifying therapies for PD.

To date, nilotinib has been the most robustly-studied rTK inhibitor for PD disease modification, and has failed to meet primary end points for disease modification, with little evidence of symptomatic benefit on several measures of PD disability [18,35]. Bosutinib, a dual inhibitor of the nRTKs cAbl and Proto-oncogene tyrosine-protein kinase Src (SRC), has shown good safety, tolerability, and target engagement in DLB, and clinically meaningful improvement in cognitive measures in AD [38,39]. Meanwhile Vodobatinib, a potent, brain-penetrant, oral c-Abl inhibitor designed to overcome nilotinib's poor CNS penetrance has not demonstrated efficacy in target engagement or disease modification in either PD or DLB [40,41]. Nevertheless, most supporting evidence for these medications to date has come from PD model systems, which have been notoriously unreliable in predicting disease modification. Our study adds to the literature by providing compelling human evidence for potential disease modification by both rTK and nrTK inhibitors, with rTK inhibitors being associated with the greatest risk reduction.

rTK expression has been shown to affect PD pathogenesis. Stable-to-increased expression of several rTKs (including platelet-derived growth factor [PDGF], EGFR, fibroblast growth factor receptor [FGFR], and VEGF) has been reported in both animal models [[44], [45], [46]] and human subjects with PD [[47], [48], [49]]. Increased expression and binding of EGFR contributes to PD neurodegeneration by promoting pro-apoptotic signaling, increasing glial reactivity, and triggering oxidative inflammatory cascades leading to neuroinflammation and cell death [50]. Furthermore, EGFR activation promotes release of proinflammatory cytokines and reactive oxygen species, exacerbating neuronal injury [44,51]. Of the medications examined in this study, only erlotinib has inhibitory action against EGFR. PDGF expression, seen in both PD and DLB, can contribute to PD-related neuroinflammation by promoting disruption and increased permeability of the blood-brain barrier through glial cell activation and amplification of proinflammatory signaling [52,53]. All rTK inhibitors examined in this study except erlotinib have some inhibitory action against PDGF. Although classically known to have neuroprotective effects in several disease states, VEGF expression and binding contributes to the neuroinflammatory changes seen in PD. In particular, VEGF promotes blood-brain barrier disruption via glial-mediated inflammatory signaling in both animal and human PD models [54]. This maladaptive angiogenic response has been linked to worsening of both motor and nonmotor symptoms [[47], [48], [49]]. Study medications with inhibitory action against VEGF include sorafenib, pazopanib, sunitinib, and nintedanib. FGFR in glia can lead to downstream release of neurotoxic mediators, amplifying glial-mediated neurotoxicity and promoting neuronal injury; ultimately, this can disrupt dopaminergic neuron homeostasis, resulting in decreased dopaminergic cell density in the substantia nigra, a key feature of PD [55,56]. Both nintedanib and pazopanib inhibit FGFR. rTK overexpression in disease states like PD may contribute to disease progression; therefore, inhibition may be protective. In addition to downregulating rTK expression, these inhibitors exhibit pleiotropic actions affecting immunomodulation, mitochondrial function, and anti-inflammatory pathways; they may even improve mitochondrial quality control, restoring parkin function and enhancing mitophagy and mitochondrial biogenesis, which are notably impaired in PD [57,58].

Several studies suggest that the nrTKs have biologic effects on pathways implicated in PD pathogenesis. The nrTK BTK, which has been demonstrated to be upregulated in PD, is correlated with serum total α-synuclein levels and is generally upregulated in a variety of neuroinflammatory and autoimmune processes [59,60]. BTK inhibition is a mechanistic feature of ibrutinib and dasatinib. SRC is a microglial activator involved in the regulation of glutamate, gamma-aminobutyric acid, and serotonin receptors, and it is positively correlated with microglial-mediated neuroinflammation [[61], [62], [63], [64]]. SRC activation and resultant inflammation are associated with neurodegenerative processes including AD and PD [65]. Of the medications examined in our study, only the multikinase inhibitor dasatinib inhibits the SRC kinase. The JAK signal transducers and activators of transcription pathway has been implicated in neurodegenerative disorders [66] and is inhibited by the JAK inhibitors ruxolitinib and tofacitinib. Several preclinical and clinical trials are examining the JAK signal transducers and activators of transcription signaling pathway in PD progression, as well as the use of JAK inhibition to reduce neuroinflammation in PD [67]. Finally, higher c-Abl activity has been observed in PD postmortem brains in the substantia nigra and the striatum, and age-dependent c-Abl activation has been observed in α-synuclein transgenic mice. The amount of c-Abl activation correlates with pathological α-synuclein accumulation, implicating a possible role of c-Abl in α-synuclein-mediated neurodegeneration [33]. Interestingly, nilotinib exhibits preferential targeting of rTK Discoidin domain receptor family, member 1 (DDR1) rather than cAbl in the context of neurodegenerative disease. DDR1, a collagen-activated rTK upregulated in PD and AD, has been implicated in neuroinflammation, dysfunction of the blood-brain barrier, impaired cellular autophagy, and cerebrovascular fibrosis. Experimental studies have shown that genetic deletion or pharmacologic inhibition of DDR1 enhances autophagic clearance of neurotoxic proteins, reduces inflammatory signaling, and improves neuropathologic outcomes. In vitro, nilotinib inhibits DDR1 at far lower concentrations than cAbl. PD trials using low-dose nilotinib demonstrated low peak CSF concentrations, which, while sufficient to inhibit DDR1, are too low to contribute to meaningful cAbl inhibition. This has shifted attention toward DDR1 as a more likely therapeutic target in neurodegeneration than cAbl. Although early clinical studies in PD, AD, and DLB have reported favorable target engagement and biomarker signals, no trial has yet demonstrated definitive clinical efficacy. Nilotinib's mechanistic role in neurodegeneration, therefore, has evolved from a c-Abl-centered model to one focused more on DDR1-mediated pro-autophagic effects. Whether sufficient CNS target engagement can be achieved to produce meaningful clinical benefit, however, remains uncertain [18,[67], [68], [69], [70], [71], [72]]. Tyrosine kinase inhibitors, while generally well tolerated, can share a common adverse effect spectrum driven by inadvertent inhibition of “off-target” kinases, which drive normal homeostatic processes. Though individual adverse effects vary widely given the breadth of indications and mechanisms, frequent class-wide toxicities can include hematologic, gastrointestinal (GI), dermatologic, cardiovascular, and metabolic abnormalities [[73], [74], [75], [76], [77], [78]].

As with any administrative data study, there are important limitations. Because Americans are not eligible for Medicare until they are 65 years of age, our study was limited to those age 65 years and older. We also restricted the study to include only Medicare beneficiaries with traditional fee-for-service Medicare, which could limit the generalizability of our findings. Additionally, we focused our analysis on a binary (yes or no) medication variable and could not examine dose response, but we did run a sensitivity analysis examining the PDC by the medication to help address this limitation. Furthermore, despite the large number of Medicare beneficiaries with PD, exposure to rTI and nrTK inhibitors of interest was uncommon, with few exposed cases and controls for individual medications. The rarity of these agents in routine clinical practice substantially reduced the effective sample size for medication-specific analyses and may have limited our ability to detect small-to-moderate associations.

An additional potential limitation of this study is confounding by indication. Many of the medications in question are prescribed for malignancies, some of which are also associated with smoking, which may independently influence PD risk. Despite this, our findings suggest that confounding by indication alone is unlikely to fully explain our observed associations. Specifically, our models adjusted for predicted probability of smoking, and in a sensitivity analysis, we adjusted for prior cancer diagnoses which did not change the findings and resulted in only modest attenuation of effect estimates. Another limitation is that we used an exposure lag of 2 years due to the limited time window of the Medicare data, which are only universally available to those 65 years of age or older. Despite this limitation, we were able to show an inverse association across all rTK inhibitors and the majority of nrTK inhibitors examined using a 2-year lag. Lastly, case misclassification is a concern when using claims data; however, we do not expect this limitation to greatly affect our results because the literature has shown that there is a high specificity in the method of PD ascertainment used in this study [79,80].

Overall, we found that use of rTK and nrTK inhibitors was associated with a lower risk of developing PD, with the greatest effects seen in association with the rTK inhibitors. Future trials of these medication classes should consider rTK inhibitors instead of nrTK inhibitors.

## Author contributions

Giovanni R Malaty: Writing – original draft; writing – review & editing; Jordan A Killion: Data curation; formal analysis; supervision, validation; writing – review & editing; Osvaldo J Laurido-Soto: Data curation (pharmacy claims); writing – review & editing; Brad A Racette: Conceptualization, funding acquisition, investigation, project administration, writing – review & editing.

## Disclosures

The authors have no personal, financial, or institutional interest in any of the drugs, materials, or devices described in this manuscript.

## Data availability statement

The data used for this manuscript is protected under a data use agreement with the Centers for Medicare and Medicaid Services and is not permitted to be distributed or shared in any format.

## Financial support

This work was supported by the US Department of Defense (PD190057); the Michael J. Fox Foundation for Parkinson's Research (MJFF-023919); Barrow Neurological Foundation; Kemper and Ethel Marley Foundation; and the Moreno Family Foundation.

## Declaration of competing interest

The authors declare that they have no known competing financial interests or personal relationships that could have appeared to influence the work reported in this paper.
